# Supplementary material for: Characterisation of the Paternal Influence on Intergenerational Offspring Cardiac and Brain Lipid Homeostasis in Mice
Source: Int J Mol Sci. 2023 Jan 17;24(3):1814. doi: 10.3390/ijms24031814 (PMC9916277; doi:10.3390/ijms24031814)
Supplement: Supplementary file 1 [file ijms-24-01814-s001.zip › Supplementary table S1.pdf]

Supplemental table S1: Normal protein diet (NPD) and low protein diet (LPD) composition

	g/kg Inclusion	
	NPD	LPD
Starch Maize	425	485
Sucrose	213	243
Corn Oil	100	100
Casein	180	90
Cellulose	50	50
Mineral mix (1)	20	20
Vitamin mix (2)	5	5
DL-methionine	5	5
Choline Chloride	2	2
Gross Energy, MJ/kg	18.39	18.27

[1] Mineral mix (AIN-76): (Special Diet Services)
[2] Vitamin mix (AIN-76): (Special Diet Services)
